# Revealing immune infiltrate characteristics and potential diagnostic value of immune-related genes in ulcerative colitis: An integrative genomic analysis

**DOI:** 10.3389/fpubh.2022.1003002

**Published:** 2022-10-31

**Authors:** Jinke Huang, Jiaqi Zhang, Fengyun Wang, Beihua Zhang, Xudong Tang

**Affiliations:** ^1^Department of Gastroenterology, Xiyuan Hospital of China Academy of Chinese Medical Sciences, Beijing, China; ^2^Institute of Digestive Diseases, Xiyuan Hospital of China Academy of Chinese Medical Sciences, Beijing, China

**Keywords:** ulcerative colitis, immune infiltration, genes, diagnostic value, genomic analysis

## Abstract

**Objectives:**

Ulcerative colitis (UC) is an autoimmune disease of the colon. The aim of this study was to explore the characteristics of immune infiltrates in UC patients and identify immune-related diagnostic biomarkers for UC.

**Methods:**

Three gene expression profiles were acquired from the GEO database, followed by identification of differentially expressed genes (DEGs) by Linear Modeling of Microarray Data. Enrichment analysis of Gene Ontology (GO), Kyoto Encyclopedia of Genes and Genomes (KEGG) and Disease Ontology (DO) were performed to analyze the biological functions of DEGs. Subsequently, the single sample gene set enrichment analysis (ssGSEA) was performed to identify immune infiltration characteristics of UC. Correlations between diagnostic genes and immune infiltration were explored to identify markers with the greatest diagnostic potential, and a UC diagnostic model was subsequently constructed. Finally, the prediction performance of the model was quantified by nomogram, non-correlated nomogram, and ROC curve.

**Results:**

A total of 3111 DEGs (1,608 up-regulated and 1,503 down-regulated genes) were identified. DEGs were significantly involved in the immune system and UC-related pathways. Immune infiltration profiles of colonic tissue were significantly different between healthy individuals and UC patients. High proportions of resting of aDCs, B cells, CD8^+^ T cells, DCs, iDCs, Macrophages, Neutrophils, pDCs, T helper cells, Tfh, Th1 cells, Th2 cells, TIL and Treg were found in UC samples. A 5-gene based diagnostic prediction model was constructed and the results of nomogram, non-correlated nomogram and ROC curve suggested the powerful diagnostic value of the model.

**Conclusions:**

This study identified the immune infiltrate characteristics and 5 immune-related genes for UC. The model based on the immune-related genes facilitates the early diagnosis of UC and provides a basis for the evaluation of the prognosis of UC.

## Introduction

Ulcerative colitis (UC) is a complex disease characterized by chronic inflammation of the colon (1). Worldwide, UC is estimated to affect 9–100,000 people annually, and the incidence is increasing year by year (2). The growing number of UC patients places a heavy economic burden on society, with direct and indirect costs ranging from $8.1–14.9 billion per year in the United States and 8012.5–29.1 billion in Europe (3). The treatment goal in UC is the induction and maintenance of remission. Although the therapeutic armamentarium is expanding, the treatment of UC is highly challenging because of its incompletely understood pathogenesis (4). Therefore, an in-depth understanding of disease pathogenesis and identification of biomarkers of disease progression at the molecular level may provide new ideas for the early diagnosis of UC.

The etiology and pathogenesis of UC are not fully understood, and it is mainly thought to be caused by an enhanced immune response to the gut microbiota in genetically susceptible individuals (5). Many studies have investigated the function of various immune cells, but it has been challenging to predict the role of all immune subsets in UC in an integrated manner. Initial activation of innate immunity causes a non-specific response, and then, sustained stimulation of inflammation will activate adaptive immunity, which may lead to persistent chronic inflammation (6). Accumulating evidence suggests that both innate and adaptive immune abnormalities are responsible for the abnormal inflammatory response in the gut (7). Inflammation associated with inflammatory bowel disease (IBD) has been reported to be closely associated with aberrant immune response elicited by CD4 T cells and dendritic cells (8–10). IRF5 contributes to the regulation of T cell signaling and modulates cytokine secretion to promote inflammation in UC (11). Neutrophil HGF-MET signaling can also contribute to the progression of UC (12). Furthermore, infiltrating immune cells are present in the intestinal mucosa of individuals with UC (13), and increased immune cell infiltration may correlate with the severity and recurrence of UC (14, 15). All these findings suggest a key role of immune cells in the pathogenesis of UC, and molecules associated with these cells may serve as new biomarkers for UC.

**Figure 1:**
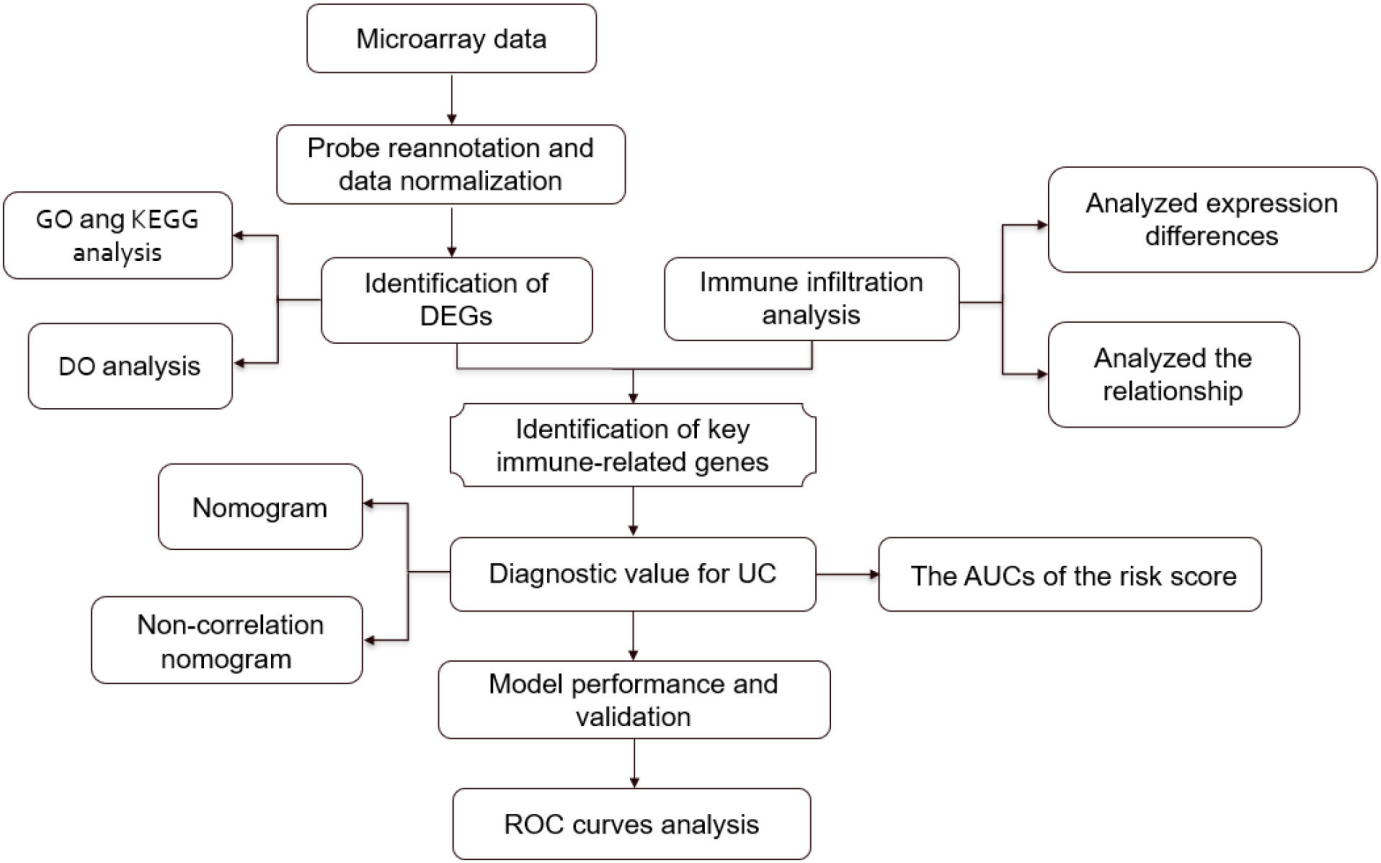
Flowchart of the present research.

Gene chip is a genetic detection technology that can detect all expression information of all genes from a sample and reveal numerous genes activated in different tissues and their physiological and pathological states (16, 17). Currently, microarray technology integrated with bioinformatics analysis has been widely used to explore pathological features and identify potential novel biomarkers for various diseases (18, 19). Based on large-scale microarray gene expression data, this study applied integrated bioinformatics analysis to explore the molecular mechanisms of UC. Moreover, we focused on identifying core genes associated with immune infiltrating cells and used these core biomarkers to construct a risk prediction model for UC with the aim of providing new ideas for early diagnosis of UC. The flow chart of the present research is shown in Figure 1.

## Materials and methods

### Microarray data acquisition

Gene expression profiles were acquired from GEO database (www.ncbi.nlm.nih.gov/geo/) (20) with the following criteria: (a) patients were diagnosed as UC; (b) data on colonic tissue from healthy controls and UC patients from the same GEO platform; (c) datasets inclusion with at least 10 UC and healthy tissue samples; (d) GEO platforms containing >5,000 genes. Finally, three gene expression profiles (GSE87473, GSE92415, and GSE 75214) were included. Table 1 shows the details of the gene expression profiles.

**Table 1:** Details of gene expression profiles.

**Dataset**	**Platform**	**Tissue**	**Normal**	**UC**	**Reference (PMID)**	**Hyperlinks**
GSE87473	GPL13158	Colon	106	21	29401083	https://www.ncbi.nlm.nih.gov/geo/query/acc.cgi?acc=GSE87473
GSE75214	GPL6244	Colon	97	11	28885228	https://www.ncbi.nlm.nih.gov/geo/query/acc.cgi?acc=GSE75214
GSE92415	GPL13158	Colon	87	21	23735746	https://www.ncbi.nlm.nih.gov/geo/query/acc.cgi?acc=GSE92415

### Identification of differentially expressed genes

After downloading the microarray expression matrices, probe names were converted to gene symbols using R software (version 4.1.2). DEGs between UC colon tissue and healthy colon tissue were screened by the “limma” package in R software, and the threshold for DEG was set to |log_2_ Fold change (FC)| > 0.5 and *p*-value < 0.05.

### Functional analysis of DEGs

Gene Ontology (GO), Kyoto Encyclopedia of Genes and Genomes (KEGG), and Disease Ontology (DO) enrichment analyses were performed on the screened DEGs from GSE87473 by the “clusterProfiler,” and “DOSE” packages in R software. The threshold for enrichment analysis was set to *p*-value < 0.05. The “ggplot2” package was applied to visualize the enrichment results.

### Immune infiltration analysis

Single sample gene set enrichment analysis (ssGSEA) method was applied to quantify the enrichment scores of immune cells (activated DCs (aDCs), B cells, CD8+ T cells, DCs, interdigitating DCs (iDCs), macrophages, mast cells, neutrophils, natural killer (NK) cells, plasmacytoid DCs (pDCs), T helper cells, T follicular helper (Tfh) cells, T helper1 (Th1) cells, T helper 2 (Th2) cells, tumor infiltrating lymphocytes (TIL), regulatory T (Treg) cells), and immune functions (antigen presenting cell (APC) co-inhibition, APC co-stimulation, chemokine receptors (CCR), check-point, cytolytic activity, human leukocyte antigen (HLA), inflammation-promoting, major histocompatibility complex (MHC) class I, Parainflammation, T cell co-inhibition, T cell co-stimulation, type-I interferon (IFN) response, type II IFN response) between control and UC group. A *p*-value < 0.05 was used to filter the samples. Heat map of the 29 types of immune cells and immune function in samples was produced by the “pheatmap” package. Levels of immune cells and immune function between UC and control samples were visualized by the “ggpubr” package. Correlative heat map was performed using the “corrplot” package to reveal the correlation of immune cells and immune function.

### The construction of diagnostic model

The top genes with the most significant differences in expression between healthy and UC samples were considered as diagnostic genes for UC, and they were evaluated by the “psych” package for their relevance to immune cell and immune function subtypes. After the feature selection, the diagnostic genes most strongly associated with immune infiltration were used to construct diagnostic models with “rms” package. The prediction performance of the model was quantified by nomogram, non-correlated nomogram, and receiver operating characteristic (ROC) curve which was performed with “ROCR” package.

### Model performance and validation

The expression data of GSE75214 and GSE92415 were used to verify the robustness of diagnostic model. The area under the curve (AUC) from a ROC curve analysis was calculated to test the diagnostic performance of the model: ROC-AUC ≥ 0.9 indicates outstanding discrimination; 0.8 ≤ ROC-AUC < 0.9 indicates excellent discrimination; 0.7 ≤ ROC-AUC < 0.8 indicates acceptable discrimination; and ROC = 0.5 indicates no discrimination (21).

### Statistical analysis

Categorical variables were presented as percentages, while continuous variables were presented as the mean ± standard deviation. All data analyses in this study were performed using R software (version 4.1.2), and the main packages that were used for t bioinformatics analysis are provided in Table 2. A *p*-value < 0.05 was considered significant for screening DEGs, enrichment analysis, correlation analysis, and immune infiltration analysis.

**Table 2:** R packages for bioinformatics analysis.

**R package**	**Version**	**Description**
Limma	3.50.3	Probe reannotation and data normalization, identification of DEGs
Pheatmap	1.0.12	Plotting heat map
Ggplot2	3.3.6	Plotting volcano plot, visualization of enrichment analysis results
Clusterprofiler	4.2.2	GO and KEGG enrichment analyses
DOSE	3.20.1	Disease ontology enrichment analysis
Corrplot	0.92	Correlation matrix visualization
Ggpubr	0.4.0	Plotting boxplot
Psych	2.2.5	Correlation analysis of DEGs and immune infiltration
RMS	6.3-0	Construction of diagnostic model
ROCR	1.0-11	ROC analysis

## Results

### Differential gene screening

**Figure 2:**
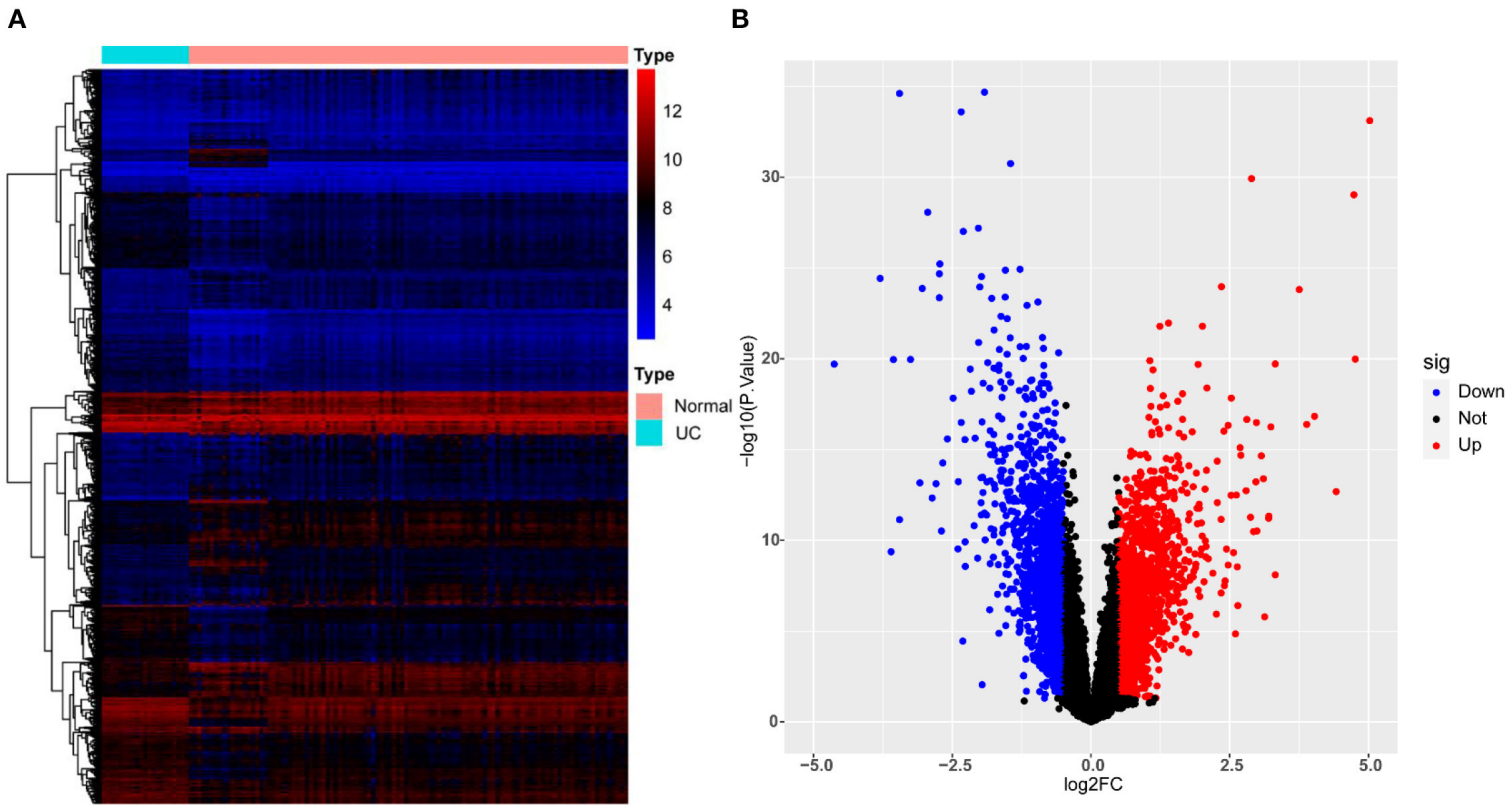
Heatmap and volcano plot for the DEGs identified from GSE87473. **(A)** Heat map for DEGs. Each row of the heat map represents one DEG, and each column represents one sample, either healthy or UC; the red and blue colors represent upregulated and downregulated DEGs, respectively. **(B)** Volcano plot for DEGs. Red plot points represent upregulated DEGs, and blue plot points show downregulated DEGs.

3111 DEGs (1,608 up-regulated and 1,503 down-regulated genes) were identified from GSE87473. The top 10 up-regulated DEGs involved were: DUOX2, MMP3, SLC6A14, DEFB4A, TNIP3, S100A8, CXCL1, DUOXA2, REG1A, and MMP10, CALU while the top 10 down-regulated DEGs were: AQP8, SLC51A, CLDN8, HMGCS2, DPP10-AS1, PCK1, ABCG2, SLC26A2, GBA3, and MEP1B. Figure 2 presents the details of the heatmap and volcano plot of DEGs.

### Functional enrichment analysis

**Figure 3:**
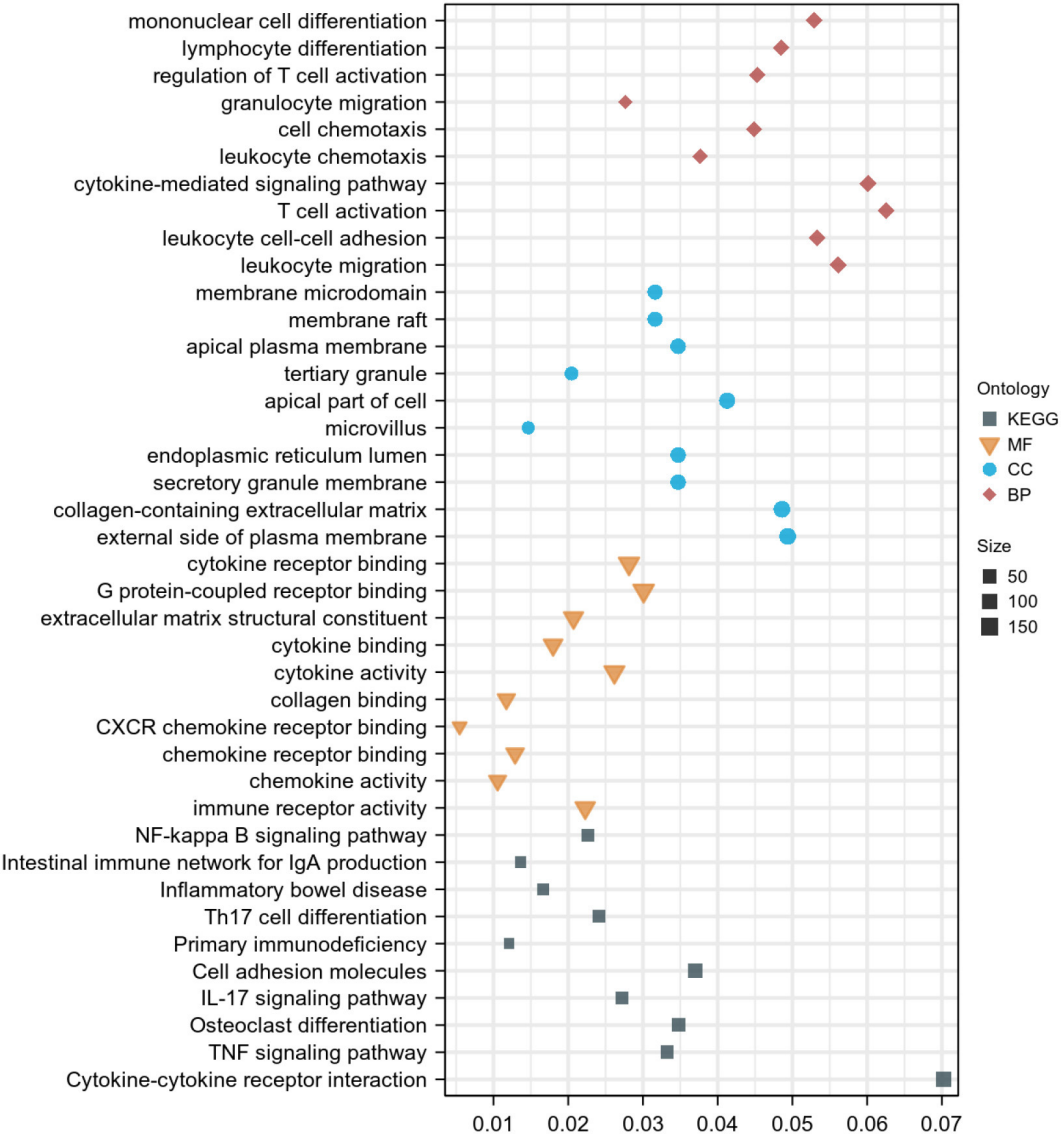
GO and KEGG enrichment analysis of DEGs. Dot graph of GO and KEGG enrichment analysis, the size of the dot represents the number of enriched genes. The figure shows the terms with *p* < 0.05.

**Figure 4:**
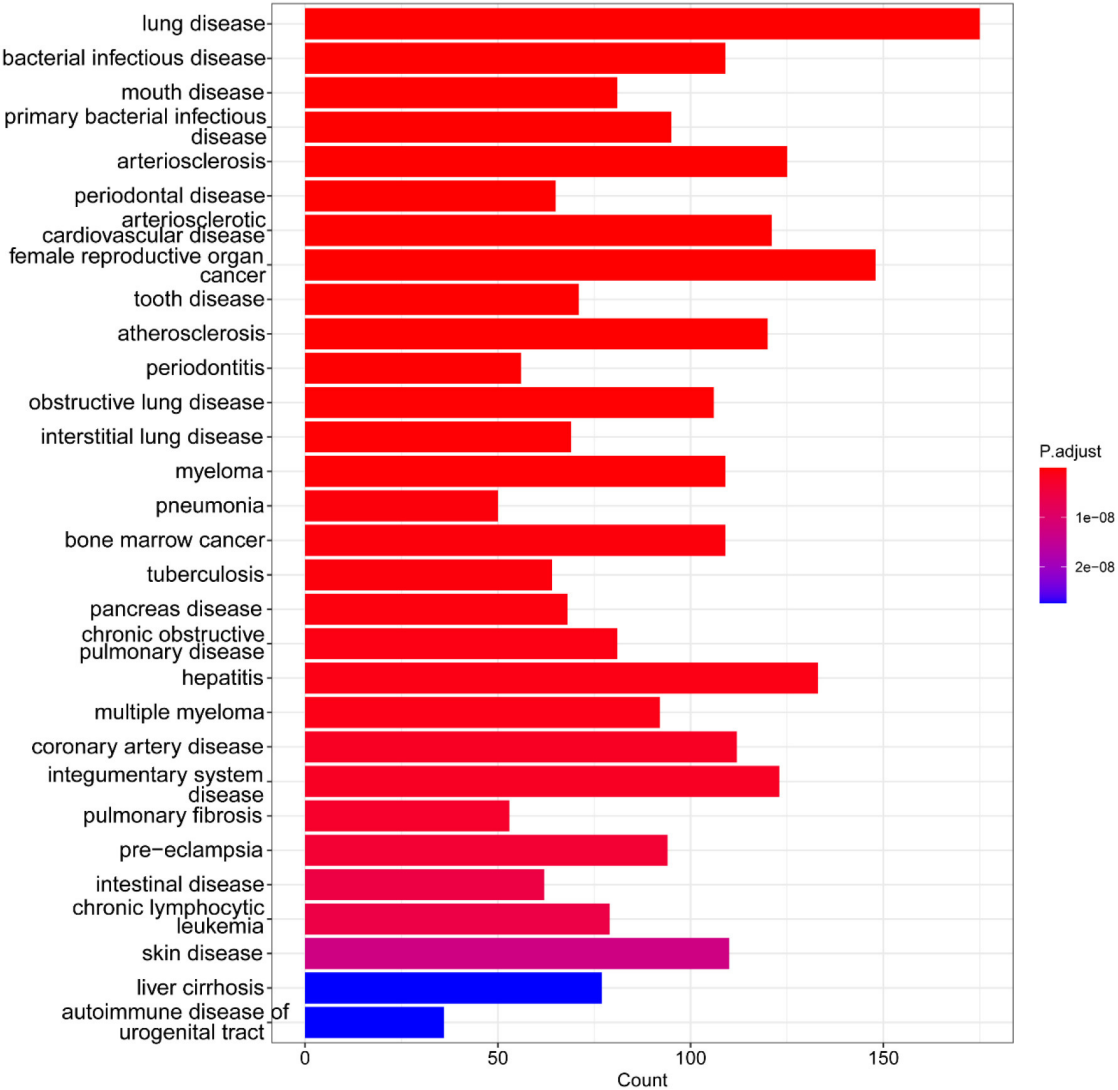
DO enrichment analysis of DEGs. Bar graph of DO enrichment analyses, the lengths of the bars represent the number of enriched genes, color represents the significance, increasing gradually from blue to red. The figure shows the terms with *p* < 0.05.

For GO analysis, DEGs were significantly enriched in the following process: leukocyte migration, leukocyte cell-cell adhesion, T cell activation, cytokine-mediated signaling pathway, leukocyte chemotaxis, cell chemotaxis, granulocyte migration, regulation of T cell activation, lymphocyte differentiation, mononuclear cell differentiation, immune receptor activity, chemokine activity, chemokine receptor binding, CXCR chemokine receptor binding, collagen binding, cytokine activity, cytokine binding, extracellular matrix structural constituent, and G protein–coupled receptor binding (Figure 3). For KEGG analysis, genes were significantly enriched in immune-related pathways such as TNF signaling pathway, Osteoclast differentiation, IL-17 signaling pathway, Th17 cell differentiation, and NF-kappa B signaling pathway (Figure 3). For DO analysis, DEGs were significantly enriched in infectious diseases, inflammatory diseases, and cancer (Figure 4).

### Immune infiltration analysis

**Figure 5:**
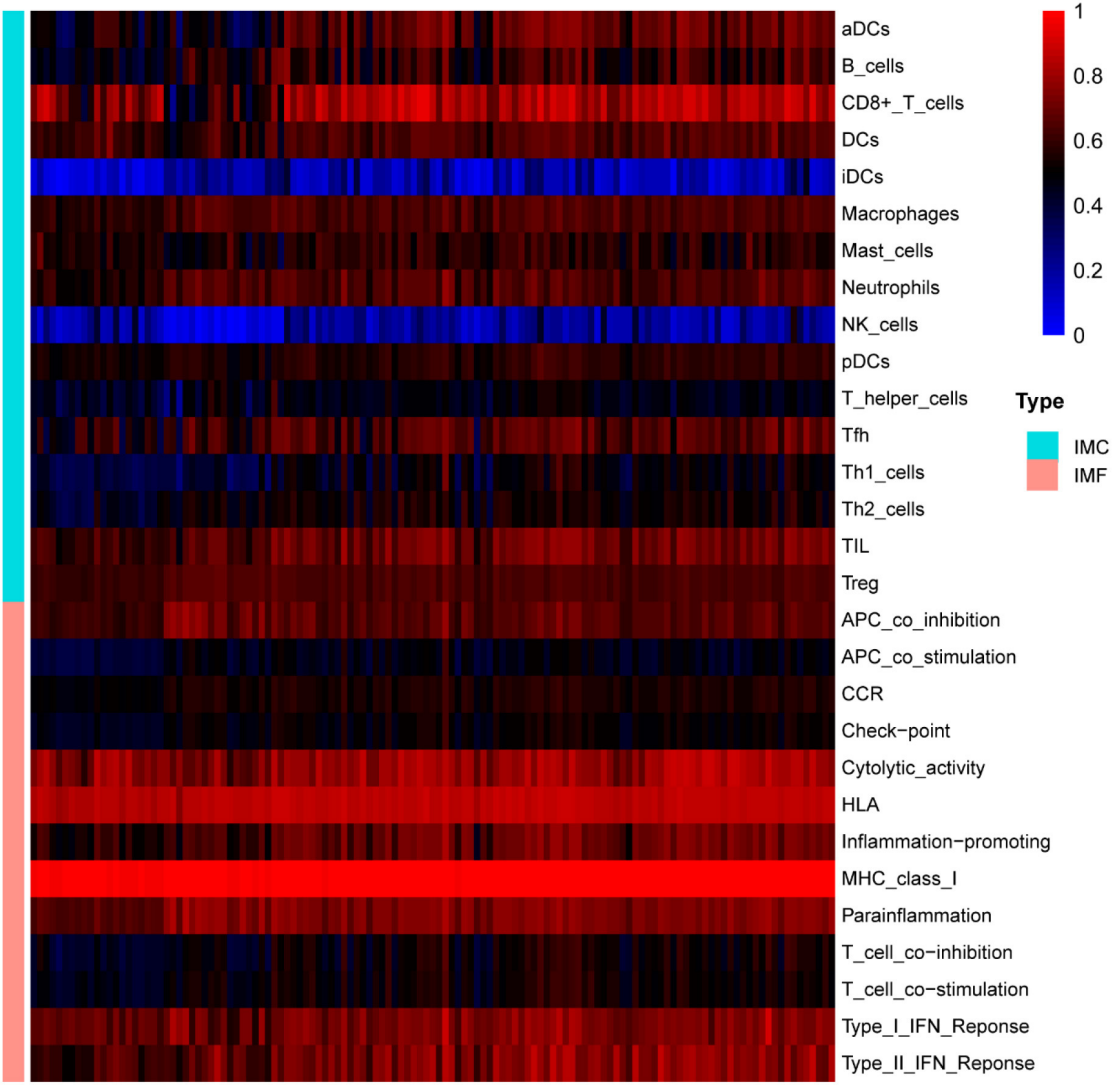
The overall landscape of immune infiltration in UC. Each row represents one sample, either healthy or UC, each column represents a type of immune cell or immune function. IMC, immune cell; IMF, immune function.

**Figure 6:**
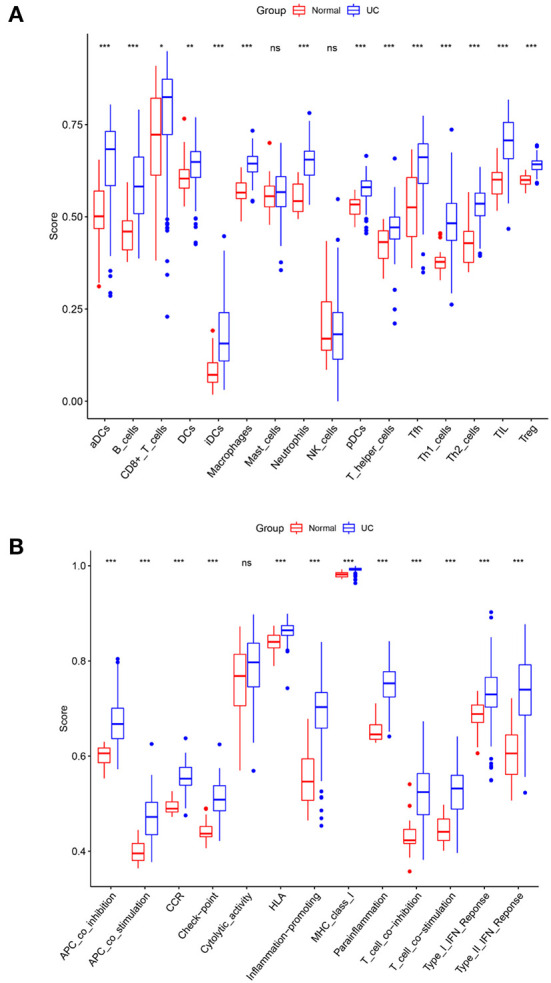
Comparison of immune cell subtypes **(A)** and immune function subtypes **(B)** between UC patients and controls. Red and blue colors represent healthy and UC samples, respectively. Differences between groups are indicated by “*”. *p < 0.05; **p < 0.01; ***p < 0.001.

The normalized enrichment score of immune infiltrates is presented in the heat map (Figure 5). The results of differential analysis of immune cell revealed that UC patients had a higher level of aDCs, B cells, CD8^+^ T cells, DCs, iDCs, Macrophages, Neutrophils, pDCs, T helper cells, Tfh, Th1 cells, Th2 cells, TIL and Treg than healthy subjects (Table 3; Figure 6A). The results of differential immune function analysis revealed that significant APC co-inhibition, APC co-stimulation, CCR, Check-point, HLA, Inflammation-promoting, MHC class I, Parainflammation, T cell co-inhibition, T cell co-stimulation, Type I IFN Reponse, and Type II IFN Reponse were observed in UC patients (Table 3; Figure 6B). Details of these biomarkers in patients with UC are presented in Table 3.

**Table 3:** Results of immune infiltration analysis.

**Immune infiltrates**	**Control**		**UC**		***P*-value**
	**Median, interquartile**	**Mean ±standard**	**Median, interquartile**	**Mean ±standard**	
	**range**	**deviation**	**range**	**deviation**	
aDCs	0.501 [0.468, 0.570]	0.495 ± 0.103	0.683 [0.584, 0.731]	0.641 ± 0.120	< 0.05
B cells	0.46 [0.410, 0489]	0.459 ± 0.058	0.582 [0.508, 0.662]	0.584 ± 0.097	< 0.05
CD8^+^ T cells	0.723 [0.613, 0.822]	0.716 ± 0.136	0.825 [0.723, 0.873]	0.775 ± 0.147	< 0.05
DCs	0.603 [0.578, 0.628]	0.608 ± 0.053	0.649 [0.607, 0.677]	0.636 ± 0.063	< 0.05
iDCs	0.072 [0.052, 0.104]	0.085 ± 0.050	0.157 [0.109, 0.240]	0.180 ± 0.091	< 0.05
Macrophages	0.566 [0.549, 0.592]	0.571 ± 0.034	0.644 [0.622, 0.664]	0.643 ± 0.037	< 0.05
Mast cells	0.556 [0.527, 0.583]	0.558 ± 0.049	0.567 [0.527, 0.609]	0.562 ± 0.067	>0.05
Neutrophils	0.543 [0.514, 0.589]	0.553 ± 0.043	0.655 [0.613, 0.678]	0.646 ± 0.050	< 0.05
NK cells	0.17 [0.138, 0.270]	0.206 ± 0.103	0.182 [0.114, 0.241]	0.185 ± 0.101	>0.05
pDCs	0.534 [0.507, 0.546]	0.531 ± 0.027	0.580 [0.557, 0.597]	0.575 ± 0.039	< 0.05
T helper cells	0.431 [0.387, 0.462]	0.424 ± 0.048	0.471 [0.440, 0.499]	0.467 ± 0.058	< 0.05
Tfh	0.526 [0.447, 0.606]	0.523 ± 0.104	0.661 [0.590, 0.698]	0.641 ± 0.084	< 0.05
Th1 cells	0.378 [0.361, 0.390]	0.381 ± 0.036	0.482 [0.436, 0.536]	0.479 ± 0.083	< 0.05
Th2 cells	0.429 [0.376, 0.460]	0.426 ± 0.057	0.535 [0.503, 0.564]	0.528 ± 0.053	< 0.05
TIL	0.601 [0.562, 0.620]	0.600 ± 0.045	0.707 [0.658, 0.756]	0.701 ± 0.068	< 0.05
Treg	0.600 [0.589, 0.611]	0.600 ± 0.015	0.642 [0.628, 0.652]	0.641 ± 0.021	< 0.05
APC co-inhibition	0.606 [0.586, 0.617]	0.601 ± 0.023	0.667 [0.637, 0.701]	0.671 ± 0.051	< 0.05
APC co-stimulation	0.396 [0.381, 0.416]	0.396 ± 0.021	0.472 [0.435, 0.503]	0.470 ± 0.045	< 0.05
CCR	0.490[0.482, 0.504]	0.492 ± 0.014	0.553 [0.539, 0.576]	0.554 ± 0.029	< 0.05
Check-point	0.437 [0.430, 0.452]	0.444 ± 0.022	0.509 [0.485, 0.538]	0.509 ± 0.038	< 0.05
Cytolytic activity	0.768 [0.706, 0.814]	0.755 ± 0.076	0.797 [0.746, 0.837]	0.787 ± 0.069	>0.05
HLA	0.840 [0.827, 0.854]	0.839 ± 0.020	0.864 [0.854, 0.874]	0.863 ± 0.020	< 0.05
Inflammation-promoting	0.546 [0.507, 0.594]	0.552 ± 0.062	0.703 [0.659, 0.733]	0.687 ± 0.075	< 0.05
MHC class I	0.982 [0.977, 0.986]	0.981 ± 0.006	0.993 [0.991, 0.995]	0.992 ± 0.006	< 0.05
Parainflammation	0.645 [0.635, 0.666]	0.651 ± 0.022	0.753 [0.724, 0.777]	0.748 ± 0.041	< 0.05
T cell co-inhibition	0.423 [0.416, 0.446]	0.434 ± 0.039	0.524 [0.477, 0.563]	0.518 ± 0.066	< 0.05
T cell co-stimulation	0.441 [0.423,0.468]	0.445 ± 0.031	0.532 [0.489, 0.560]	0.526 ± 0.052	< 0.05
Type I IFN response	0.689 [0.671, 0.707]	0.682 ± 0.038	0.730 [0.703, 0.766]	0.729 ± 0.064	< 0.05
Type II IFN response	0.606 [0.561, 0.644]	0.608 ± 0.063	0.740 [0.686, 0.792]	0.734 ± 0.080	< 0.05

**Figure 7:**
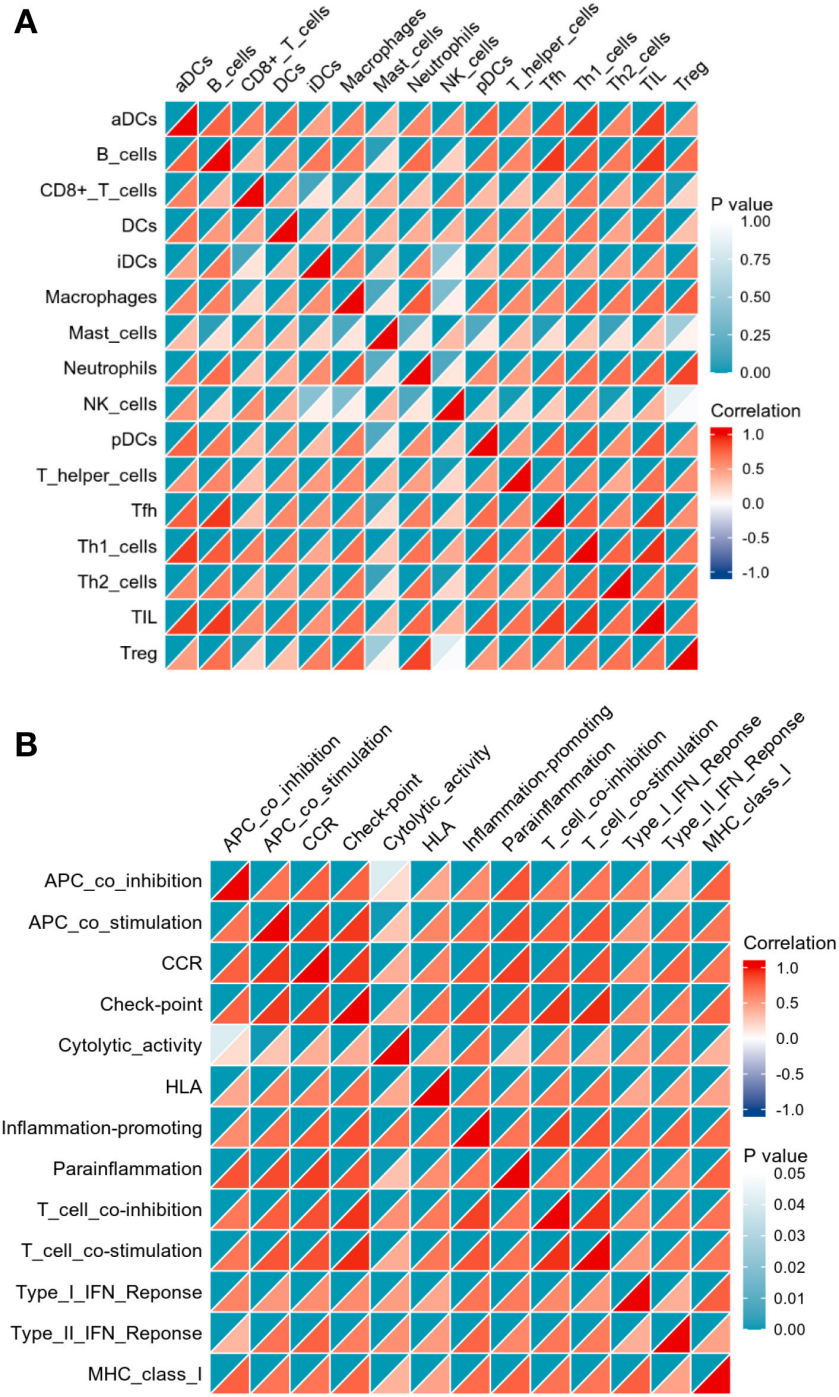
Correlation matrix of immune cell subtypes **(A)** and immune function subtypes **(B)**. Both horizontal and vertical axes demonstrate immune cell subtypes and immune function subtypes (blue for negative correlation, white for weak or no correlation), red for positive correlation). Color represents the significance, increasing gradually from white to indigo.

The correlation analysis revealed that activated B cells were not related to Mast cells; CD8^+^ T cells were not related to iDCs; iDCs were not related to NK cells; Macrophages were not related to Mast cells or NK cells; Mast cells were not related to Neutrophils, pDCs, Th2 cells or Treg; and NK cells were not related to Treg (Figure 7A). However, strong correlations were observed for all other types of immune cells and immune function (Figure 7).

### The construction of diagnostic model

**Figure 8:**
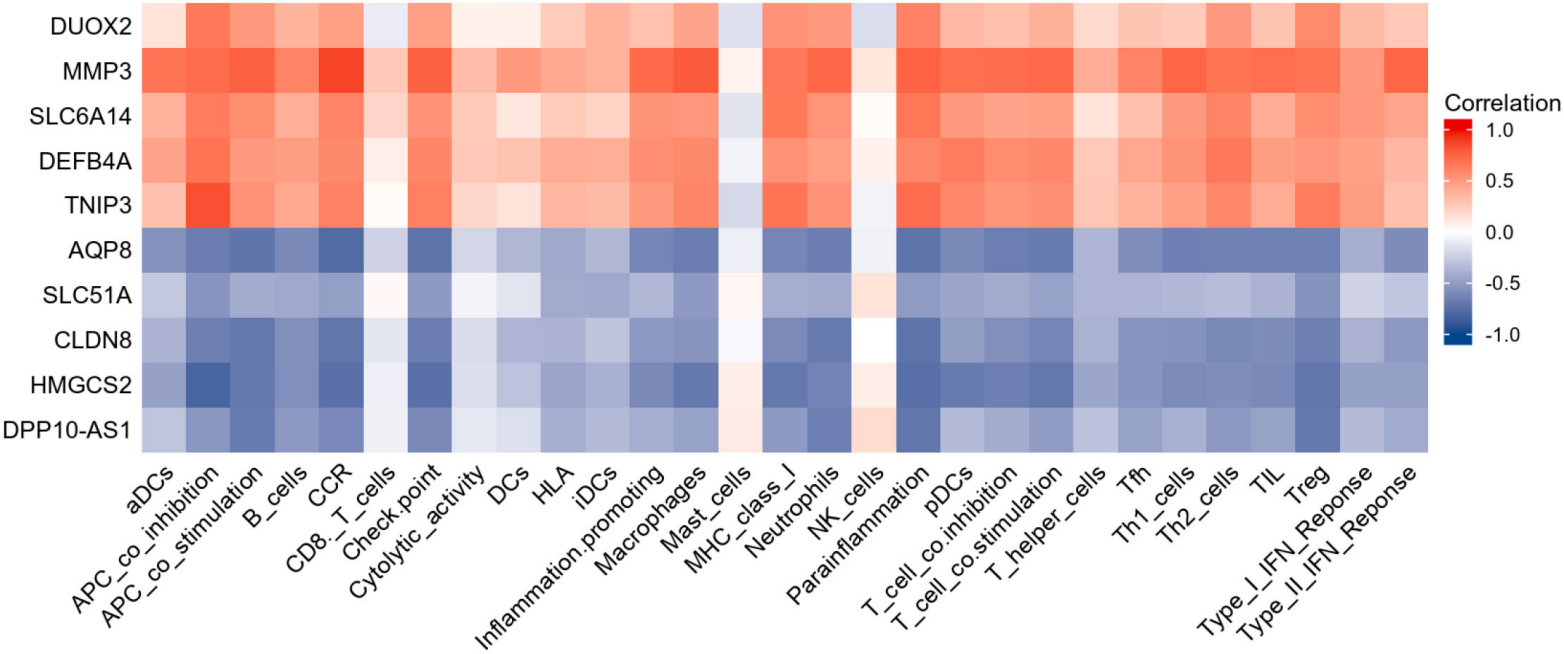
Heatmap showing the relationship between candidate genes and immune infiltrate. Red is positive correlation and blue is negative correlation.

Results of Pearson correlation analysis revealed that all upregulated diagnostic genes were significantly positively correlated with almost all immune cell subtypes and immune function subtypes (except CD8^+^ T cells and NK cells). Similarly, almost all down-regulated diagnostic genes were negatively correlated with almost all immune cell subtypes and immune function subtypes (except CD8^+^ T cells and NK cells) (Figure 8).

Inspired by the above results, five diagnostic genes most associated with immune infiltration (HMGCS2, CLDN8, AQP8, DEFB4A, MMP3) were used to construct a diagnostic model for UC. Details of these biomarkers in patients with UC are presented in Table 4.

**Table 4:** Details of genes in diagnostic model.

**Genes**	**Sig**	**Log2FC**	**Average expression**
AQP8	Down	−4.626352947	8.071990976
CLDN8	Down	−3.601376685	6.380647165
HMGCS2	Down	−3.559635503	7.584732929
DEFB4A	Up	4.418274083	8.68987552
MMP3	Up	4.763273249	9.167130008

**Figure 9:**
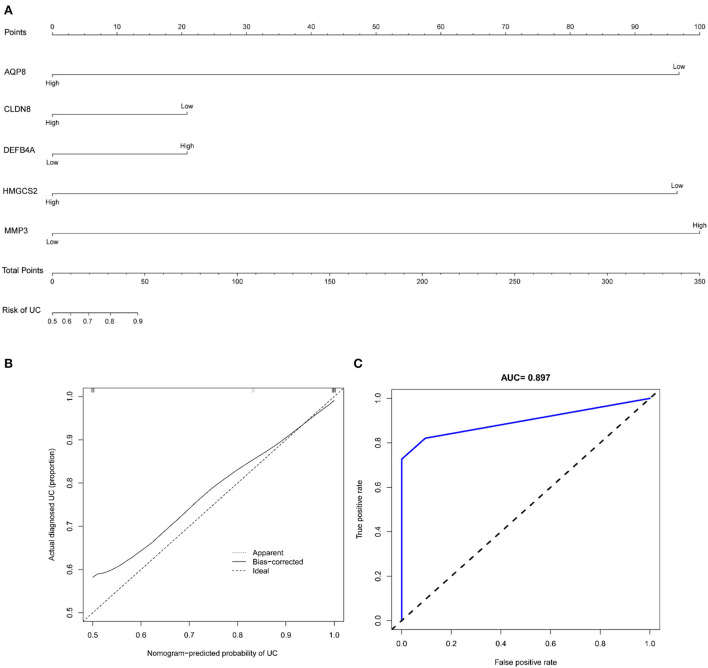
**(A)** Nomogram of diagnostic marker genes; **(B)** calibration curve of non-correlation nomogram prediction in the cohort; **(C)** ROC curve for the diagnostic efficacy of diagnostic model.

The nomogram showed the diagnostic efficacy of the model constructed with these predicted diagnostic genes for UC (Figure 9A). Based on the calibration curve predicted by the uncorrelated nomogram, the performance of the column line plot was close to the ideal model, suggesting that the predictive value of the model is credible (Figure 9B). Similarly, ROC-AUC of the risk score was 0.897, which indicates excellent discrimination of the model (Figure 9C).

### Model performance and validation

**Figure 10:**
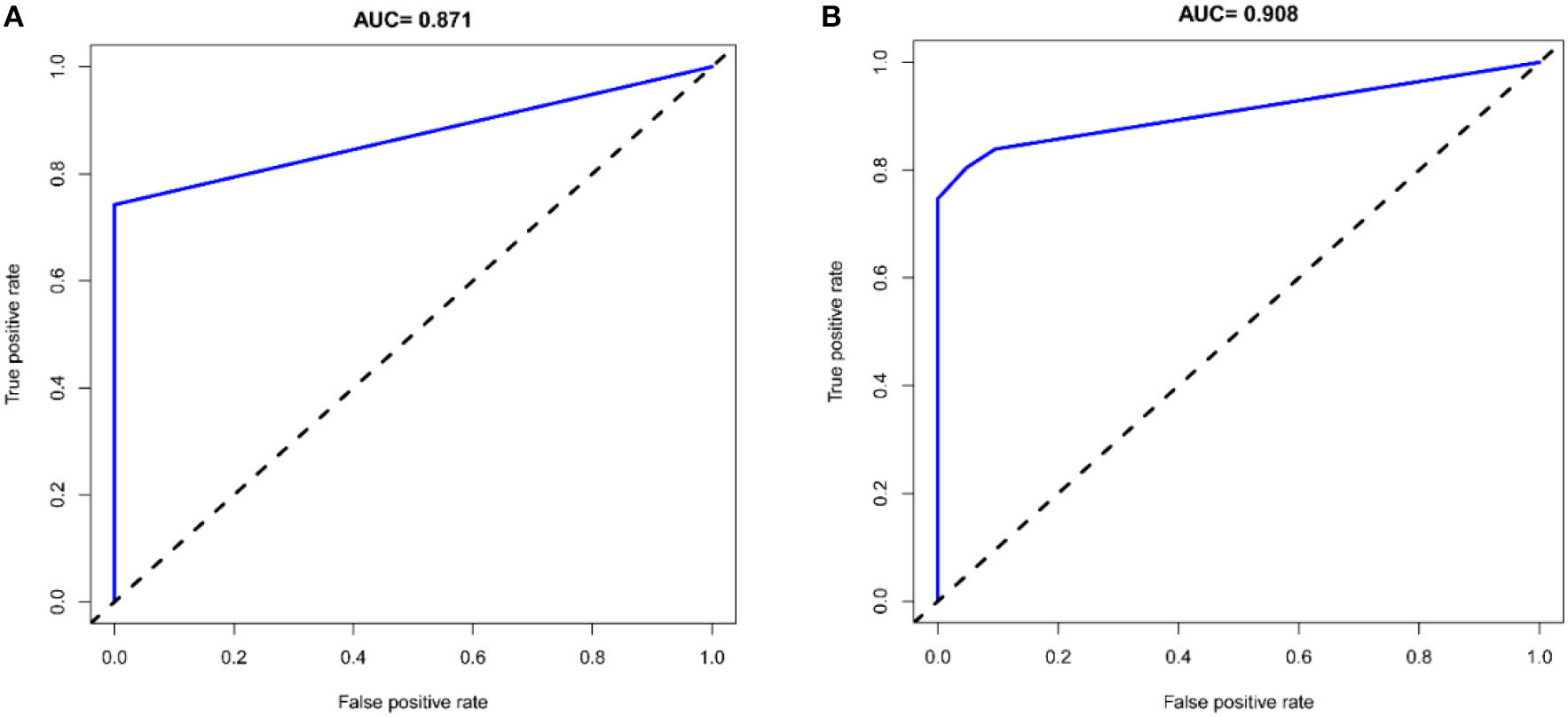
ROC curves for the model in the validation dataset of GSE75214 **(A)** and GSE92415 **(B)**.

To go step further validation, ROC curves were applied to assesses the prediction accuracy of the model. The ROC-AUC of the risk score was 0.871 in GSE75214 and 0.908 in GSE92415, respectively, indicating excellent discrimination of the model (Figure 10).

## Discussion

The development of UC involves genetic susceptibility, environmental factors and disturbances in the gut microbiota and is characterized by an abnormal mucosal immune response and a persistent inflammatory infiltrate. Pathological mechanisms that have not been fully elucidated have limited the development of early diagnosis and treatment of UC. Currently, integrated microarray-based bioinformatics analysis has been widely used to identify potential novel biomarkers for various diseases, which is important for specifying molecular markers and diagnosing UC early in the disease process. In this study, DEGs were identified to be mainly enriched in immune response-related pathways. Immune infiltration analysis suggested significant differences in immune cell and immune function types between UC patients and controls. Correlation analysis of DEGs and immune microenvironment was performed, and five immune-related genes (HMGCS2, CLDN8, AQP8, DEFB4A, MMP3) were identified and were subsequently used to construct a diagnostic model of UC. Encouragingly, this model showed good diagnostic performance for UC, and data from the other two datasets further validated the accuracy of this model for UC diagnosis.

With data from GEO, 3111 DEGs (1,608 up-regulated genes and 1,503 down-regulated genes) were obtained in UC by comprehensive analysis of the microarray dataset (GSE87473). Further GO analysis indicated that these DEGs were significantly enriched in the leukocyte migration, leukocyte cell-cell adhesion, T cell activation, cytokine-mediated signaling pathway, leukocyte chemotaxis, cell chemotaxis, granulocyte migration, regulation of T cell activation, lymphocyte differentiation, mononuclear cell differentiation, immune receptor activity, chemokine activity, chemokine receptor binding, CXCR chemokine receptor binding, collagen binding, cytokine activity, cytokine binding, extracellular matrix structural constituent, and G protein–coupled receptor binding. For KEGG analysis, genes were significantly enriched in immune-related pathways such as TNF signaling pathway, Osteoclast differentiation, IL-17 signaling pathway, Th17 cell differentiation, and NF-kappa B signaling pathway. For DO analysis, DEGs were significantly enriched in lung disease, intestinal disease, mouth disease, primary bacterial infectious disease, arteriosclerosis, IBD, arteriosclerotic cardiovascular disease, female reproductive organ cancer, and tooth disease. In summary, results of the bioinformatics analyses suggested that these DEGs were closely related to immune cell infiltration in UC. These findings further validate the key role of immune abnormalities in the pathological progression of UC (22).

Inspired by the results of functional analysis of DEGs, immune infiltration analysis was further performed. The results revealed that there were significant differences in aDCs, B cells, CD8^+^ Tcells, DCs, iDCs, Macrophages, Neutrophils, pDCs, T helper cells, Tfh, Th1 cells, Th2 cells, TIL and Treg between colon tissue in UC patients and the healthy group. In fact, most of the above immune cells have been reported to be in an abnormal state in UC by previous studies (23, 24). Therefore, these identified immune cells may be involved in the development and progression of UC. Top genes with the most significant expression differences between healthy and UC samples were considered as potential diagnostic genes for UC. After feature selection, HMGCS2, CLDN8, AQP8, DEFB4A, and MMP3 were identified as having strong association with immune infiltration, and thus they may be key genes that identify the immune features of UC and are involved in UC immune regulation.

Inspired by the results of functional analysis of DEGs and immune infiltration analysis, HMGCS2, CLDN8, AQP8, DEFB4A, and MMP3 were applied to construct a diagnostic model for UC. The nomogram showed the well diagnostic efficacy of the model constructed with these predicted diagnostic genes for UC. The calibration curve for the uncorrelated nomogram prediction showed that the performance of the column line plot was close to the ideal model, suggesting that the predictive value of the model is credible. Similarly, ROC-AUC of the risk score was 0.897, suggesting a high diagnostic efficiency of the diagnostic marker gene model. To go step further validation, data from GSE75214 and GSE92415 were applied to assesses the prediction accuracy of the model. The results revealed that ROC-AUC of the risk score were 0.871 in GSE75214 and 0.908 in GSE92415, respectively, indicating excellent discrimination of the diagnostic model.

A total of five immune-related genes were included in the diagnostic model. The protein encoded by HMGCS2 belongs to the HMG-CoA synthase family of mitochondrial enzymes that catalyze the first reaction of ketogenesis, which is a crucial alternative metabolic pathway and is involved in the regulation of the body's immune function (25). Restoration of ketogenesis enhances immune cell effects (26) and attenuates the activation of pro-inflammatory macrophages (27). The protein levels of HMGCS2 in the intestinal epithelium of UC patients were reported to be sharply decreased compared to healthy samples (28). Increased ketogenesis may help to counteract intestinal inflammation, and conversely, its suppression may exacerbate intestinal pathology (28). The CLDN8 gene encodes a member protein of the claudin family. Claudins are integral membrane proteins and components of tight junction chains that play a key role in maintaining the integrity of the intestinal mucosal barrier. CLDN8 was reported to be significantly downregulated in the biological colon of IBD patients, and similar results were observed in colitis mice (29). AQP8 encodes an epithelial water transport protein specifically expressed in colonic absorptive cells, and it was found to be significantly downregulated in UC patients compared to healthy controls (30, 31). In addition, AQP8 was observed to promote H_2_O_2_ diffusion in experimental mouse models, which suggested its balance and regulatory effects on antioxidant pathways (32). DEFB4A encodes defensin, beta 4, an antibiotic peptide locally regulated by inflammation, which has been shown to be involved in the pathological process of IBD. Results of high-throughput sequencing suggested that the composition of the microbiota differs significantly between UC and non-IBD. Alterations in the microbiota can affect antimicrobial peptide expression, which in turn is involved in the progression of IBD (33). Various studies have highlighted the involvement of specific matrix metalloprotease in IBD: MMP3 transcript or protein levels are upregulated in the mucosa of inflammatory IBD or in the serum of IBD patients, and MMP protein hydrolytic activity is increased in epithelial-derived cells of inflammatory IBD (34–36). It was found that MMP3 expression was significantly upregulated in inflammatory colonic segments of IBD patients compared to non-inflammatory regions (37). In addition, MMP3 serum assay possesses a suggestive role for early response to infliximab treatment of UC (38). In summary, these key genes are all involved in the development of UC, and their inclusion in our diagnostic model of UC is reasonable.

Although this study applied a relatively large sample size to characterize the immune microenvironment and construct a diagnostic model for UC by integrating the GEO dataset, limitations should be acknowledged. First, this study explored the infiltration of immune cells by ssGSEA and found that immune cells play an important role in the pathological progression of UC. Therefore, it is crucial to validate our findings by flow cytometry. Second, although the present model may serve as a valid predictive tool for UC diagnosis, the true predictive value of the model should be prospectively validated in future independent and multicentered-studies with larger sample sizes.

## Conclusion

In conclusion, this study identified the immune infiltrate characteristics and five immune-related genes for UC. The model based on the immune -related genes facilitates the early diagnosis of UC and provides a basis for the evaluation of the prognosis of UC.

## Data availability statement

The original contributions presented in the study are included in the article/Supplementary material, further inquiries can be directed to the corresponding author/s.

## Author contributions

JH drafted the manuscript. JZ, FW, and BZ helped with implementation of this work. XT contributed to the methodology, review, and editing of the manuscript. All authors read and approved the final manuscript.

## Funding

This work was supported by the National Natural Science Foundation of China (No. 81830118), China Academy of Chinese Medical Sciences Innovation Fund (No. CI 2021A01012), China Academy of Chinese Medical Sciences Excellent Young Talent Cultivation Fund (No. ZZ 15-YQ-002), and Administration of Traditional Chinese Medicine Digestive Refractory Disease Inheritance and Innovation Team Project (No. ZYYCXTD-C-C202010).

## Conflict of interest

The authors declare that the research was conducted in the absence of any commercial or financial relationships that could be construed as a potential conflict of interest.

## Publisher's note

All claims expressed in this article are solely those of the authors and do not necessarily represent those of their affiliated organizations, or those of the publisher, the editors and the reviewers. Any product that may be evaluated in this article, or claim that may be made by its manufacturer, is not guaranteed or endorsed by the publisher.
