# Supplementary figures and images for: Revealing immune infiltrate characteristics and potential diagnostic value of immune-related genes in ulcerative colitis: An integrative genomic analysis
Source: Front Public Health. 2022 Oct 31;10:1003002. doi: 10.3389/fpubh.2022.1003002 (PMC9660254; doi:10.3389/fpubh.2022.1003002)

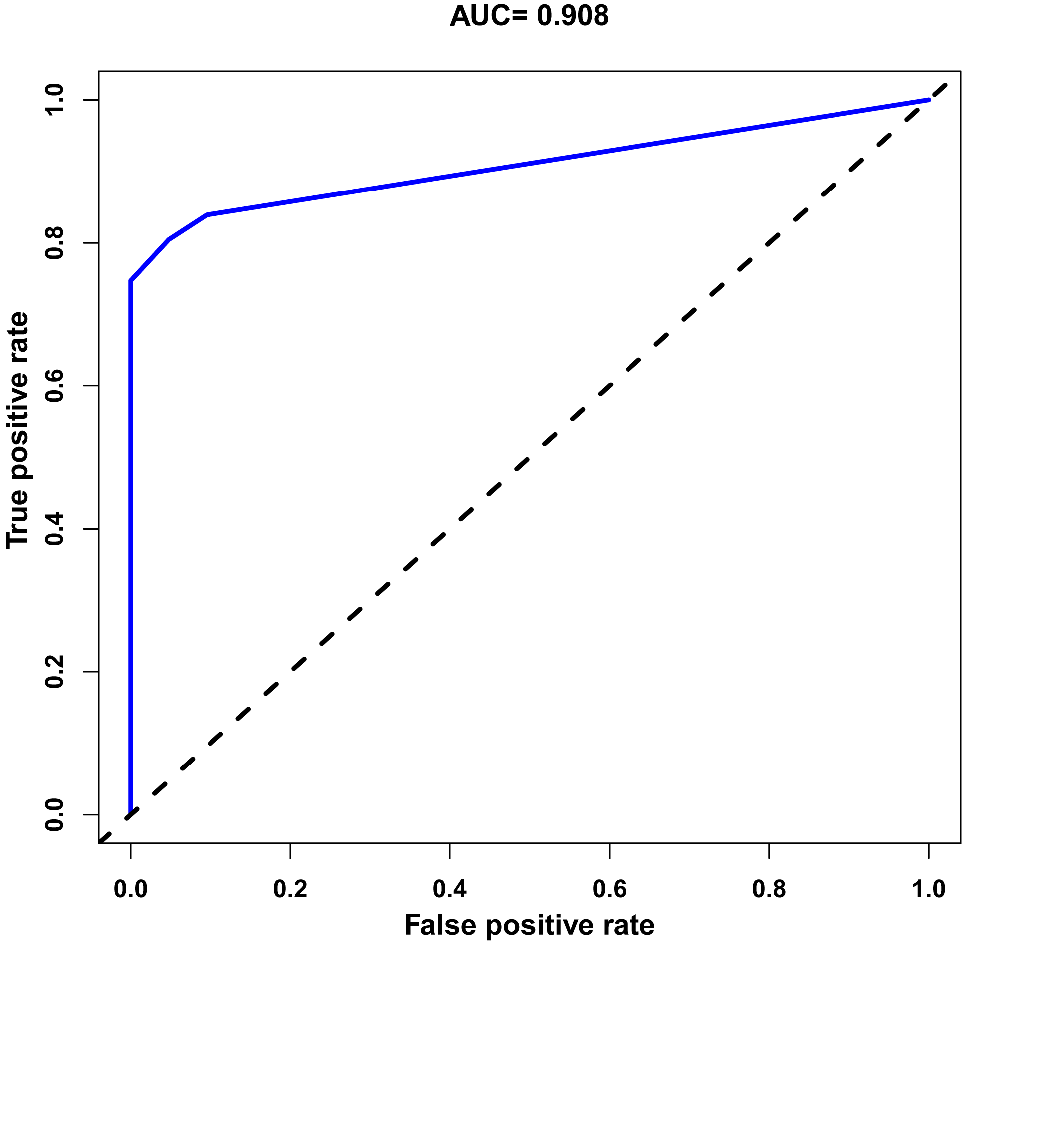

Supplement: Supplementary file 1 [file Image_1.TIF]
